# Copolymerization of UF Resins with Dimethylurea for Improving Storage Stability without Impairing Adhesive Performance

**DOI:** 10.3390/ma11061032

**Published:** 2018-06-19

**Authors:** Pedro Pereira, João Pereira, Nádia. T. Paiva, João. M. Ferra, Jorge M. Martins, Luísa. H. Carvalho, Fernão. D. Magalhães

**Affiliations:** 1 EuroResinas-Indústrias Químicas, 7520 Sines, Portugal; pmpereira352@gmail.com (P.P.); nadia.paiva@sonaearauco.com (N.T.P.); joao.ferra@sonaearauco.com (J.M.F.); 2 ARCP—Associação Rede de Competência em Polímeros, 4200 Porto, Portugal; jamp@fe.up.pt; 3 DEMad—Instituto Politécnico de Viseu, 3504 Viseu, Portugal; jmmartins@estv.ipv.pt (J.M.M.); lhcarvalho@demad.estv.ipv.pt (L.H.C.); 4 LEPABE—Faculdade de Engenharia da Universidade do Porto, 4200 Porto, Portugal

**Keywords:** urea-formaldehyde resins, storage stability, copolymerization, dimethylurea

## Abstract

Urea-formaldehyde (UF) resins are the most used resins in the wood industry due to high reactivity and low price. However, their reduced stability during storage is a drawback, imposing strict limits in terms of allowable shipping distances and storage times. This instability, manifested by viscosity increase that renders the resin unusable, occurs due to the progress of condensation reactions between the polymeric species present in the liquid medium. In order to achieve a stable resin formulation, dimethylurea (DMeU) was selected for being less reactive than urea. Dimethylurea is shown to co-polymerize with the UF polymer during the acidic synthesis condensation step. However, during storage it behaves like an end group blocker, due to its lower reactivity at basic pH. By adding 1.25% DMeU, it was possible to obtain a formulation that remained with stable viscosity during two-month storage at 40 °C. The reference UF resin remained stable only for eight days in these conditions. Wood particleboards produced with modified resins showed internal bond strengths of about 0.5 N·mm^−2^, similar to the fresh reference UF resin, even when the resins were used after the two-month storage period. Formaldehyde content values were below the limit for E1 class, ≤8 mg/100 g oven dry board (EN 13986).

## 1. Introduction

Urea-formaldehyde (UF) resins are widely used in the production of particleboard (PB) and medium-density fiberboard (MDF), presenting good properties like high reactivity, high bond strength, water dispersibility, and low-cost [1]. However, just like all amino resins, they have relatively low storage stability: about 1 month at 25 °C, and much lower if subjected to higher temperatures. This is a serious limitation when long-distance transportation or relatively long-term storage are intended. Previous works have focused on the physico-chemical processes that take place during storage, but no information exists on how to improve stability [2,3].

The instability of UF resins is caused by the progress of poly-condensation reactions, resulting in an increase in resin viscosity [2,4]. Condensation reactions are preferably promoted by acidic medium; however, they may also occur at a slower rate in basic medium [5]. They involve reactions between primary or secondary amine groups on the end groups of the polymer, which act as nucleophilic groups, and hidroxymethyl groups present along the polymer structure, which act as electrophilic groups due to the adjacent carbon. Another possible condensation reaction involves two hidroxymethyl groups, since they can simultaneously act as electrophilic and nucleophilic groups, which are the hydroxyl group [6,7]. Condensation reactions also take place between monomers and polymer, however their contribution to the viscosity increase is less relevant.

In a previous studies, we have observed that the presence of monomers at the end of synthesis influences negatively the pH, and thus the storage stability of the resin. It was also observedthat adding a monofunctional monomer, to block a fraction of the reactive sites, together with insuring basic pH during storage, to lower the rate of polycondensation reactions, is an effective strategy for significantly improving storage stability. However, this compromises the physico-mechanical properties of the wood particleboards produced with the modified resins, resulting in high amounts of free formaldehyde and lower internal bond strength. Therefore, a more effective alternative may be to use a comonomer that acts as a blocker during storage, but allows polymer crosslinking in the curing process. For that purpose, dimethylurea (DMeU) was chosen in this work. It has a structure similar to urea, with two amino groups, but it is much less reactive, especially at basic pH, which coincides with storage conditions. This lower reactivity is related with the type of amines present in each compound, since primary amines (urea) are more reactive than secondary amines (DMeU). Besides that, secondary amines only have two possible sites for reaction while urea has four. It is expected that DMeU can react with the UF polymer during the synthesis process, inserting a low reactivity end group that will not participate in condensation reactions during storage. Under curing conditions (high temperature and acidic pH), the DMeU end group should enable crosslinking. Existing studies show that DMeU is able to react with formaldehyde, proving that it can participate in condensation reactions during resin synthesis [8]. The goal of this work is to evaluate dimethylurea effectiveness in obtaining a modified UF resin that remains stable during storage for two months under a relatively high temperature (40 °C), without having a negative impact on the resin’s performance.

## 2. Materials and Methods

### 2.1. Materials

The following industrial-grade reagents were supplied by Euroresinas S.A. (Sines, Portugal): urea, formaldehyde 55 wt %, sodium hydroxide 50 wt % and acetic acid 25 wt %. Sodium bicarbonate was purchased from Sigma–Aldrich (St. Louis, MO, USA) and *N*,*N*′ dimethylurea 98% was purchased from Alfa Aesar (Haverhill, MA, USA). The chemicals were used as received without further purification.

Wood particles were provided by a particleboard manufacturer (Sonae Arauco, Oliveira do Hospital, Portugal).

### 2.2. Synthesis of UF Resins

The UF resins were synthesized according to the alkaline-acid synthesis process, as previously described [9]. A round bottom flask (volume 2 L) was used, equipped with mechanical stirrer, water cooled condenser, and a thermometer. Formaldehyde solution (50 wt % aqueous solution) and the first urea were added to obtain an formaldehyde/urea (F/U) molar ratio of at least 3, followed by sodium hydroxide (50% (m/m)) to adjust the pH to a value between 8.0 and 9.0. This methylolation step lasted 30 min at 75–90 °C. The pH was then adjusted to between 5.0 and 6.5 in order to start the condensation step. The second urea was added at this point to obtain an F/U ratio between 1.6 and 2.6. When the viscosity reached the limit value of 200–350 mPa∙s, sodium bicarbonate was added to stop the condensation step by lowering the pH and cooling the reaction mixture. Before the characterization and analysis of the resin’s mechanical properties, the third and final urea was added to the formulation in order to obtain a molar ratio between 1.10 and 1.15. This product will be referred to as the reference resin (REF).

Dimethylurea was added at two distinct times. In one case, DMeU was added right subsequently to the second urea, at the beginning of the condensation step under acid pH. In the other, DMeU was added after the end of condensation, after the pH was lowered and at a temperature of 40–50 °C. The percentage of DMeU added is related to the total solid content in the final resin (i.e., after final urea addition). 

### 2.3. Resin Characterization

Viscosity, pH, gel time, and solid content were determined at the end of each synthesis. Viscosity was measured with a Brookfield viscometer, using spindle number 62 at a rotational speed of 60 rpm. The resin pH was measured using a combined glass electrode. The resin gel time was determined by measuring the time needed for resin gelification at 100 °C, after addition of a cure catalyst (ammonium sulphate). The solid content was determined by evaporation of volatiles from 2 g of resin for 3 h at 120 °C. In order to evaluate the stability of the resins, they were stored in an incubator at 40 °C. The viscosity was periodically measured with a Brookfield DVII+ viscometer (Brookfield, Toronto, ON, Canada) at the same temperature. It was confirmed that the liquid temperature did not drop by more than 1 °C during the viscosity measurements.

### 2.4. ^13^C NMR Spectroscopy

The samples were prepared with a weight of about 50 mg of resin and completed with 0.75 mL of DMSO-*d*_6_. The spectra were obtained on a Bruker Avance III 400 NMR spectrometer (Billerica, MA, USA) using a repetition delay of 10 s. In order to obtain a quantitative analysis, spectra were accumulated with 3200 scans. The peak areas determined were presented in percentages to allow the comparison of the two resins.

### 2.5. Formaldehyde Content of the Resins

Formaldehyde analysis were performed to determinate the percentage of free formaldehyde according to the [10]. First, the resin was added to a 50/50% solution of DMSO/H_2_O. Then an acid solution of 0.1 M was added, which contained sodium sulphite, followed by the titration with a sodium hydroxide solution of 0.1 M. During the process the solution with the resin was at 0 °C. 

### 2.6. Automated Bonding Evaluation System (ABES)

Preliminary resin bond ability tests were performed with ABES (Adhesive Evaluation Systems, Inc., Corvallis, OR, USA), in order to establish the pressing conditions [11]. Two beech veneer strips were used, each measuring 0.5 mm thick, 20 mm wide, and 117 mm in length. The glue mix was applied manually with a spatula (6 mg) and the spread rate (100 g/m^2^) was controlled in a precision balance. The trial conditions were 3% of catalyst and at a temperature of 105 °C. The trial was made as described in Reference [12].

### 2.7. Particleboard Production

The production of particleboard was essentially divided into four stages: preparation of raw materials, blending, mat formation, and pressing. Standard mixtures of wood were used for the core and face layers, which are composed of different proportions of pine, eucalyptus, pine sawdust, and recycled wood. The moisture content of the standard mixtures was checked before blending, using an infrared balance. Wood particles were then blended with the resin, catalyst, and paraffin in a laboratory glue blender. The gluing factor was 6.0% resin solids in both layers, based on the oven-dry weight of wood particles. The amount of ammonium sulfate was 1% (based in solid resin) in face layer and 3% (based in solid resin) in core layer. The amount of paraffin was 2% (based in solid resin) in face and core layer. Particleboards were prepared in an aluminum container with 220 × 220 × 80 mm^3^ and were structured in three layers: upper face layer (20%), core layer (62%), and bottom face layer (18%). Then, they were pressed in a computer-controlled laboratory hot-press at 190 °C, with pressing times of 120 s and 150 s. The average density of the final boards was (630 ± 20) kg·m^−3^. 

After pressing, boards were stored in a conditioned room (20 °C, 65% RH) and tested accordingly to European Standards. The following physico-mechanical properties were evaluated: density [13], moisture content [14], internal bond strength (IB) [15], thickness swelling [16], and formaldehyde content [17]. For each experiment, three board replicates were obtained.

## 3. Results

This section may be divided by subheadings. It should provide a concise and precise description of the experimental results, their interpretation, as well as the experimental conclusions that can be drawn.

### 3.1. Incorporation of DMeU at Different Stages

Dimethylurea was added in two different stages of the synthesis process: in the beginning of the condensation step and after synthesis. Dimethylurea was expected to react with the polymer only in the first case, where high temperature and acidic pH favor reaction between monomers. In the second case, DMeU should not become incorporated in the polymer, being only present in the aqueous phase. The amount of DMeU added was 5% of the total mass of resin. To simplify the discussion, the resin where DMeU was added in the condensation step will be called resin IC and the resin where DMeU was added after condensation is called AC.

**Figure 1:**
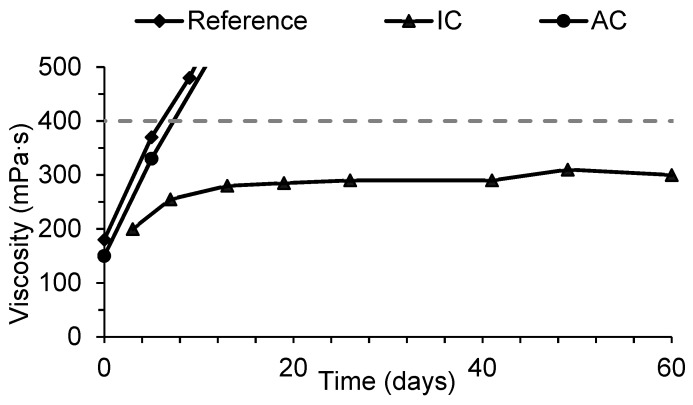
Viscosity as a function of storage time at 40 °C for the reference resin and resins with 5% of dimethylurea added at different phases. The viscosity limit considered for stability is shown as a dashed horizontal line. Resin where Dimethylurea (DMeU) was added in the condensation step = IC, resin where DMeU was added after condensation = AC.

Figure 1 presents the evolution of viscosity along storage time for each resin. The limiting viscosity value considered acceptable is 400 mPa∙s at 40 °C. When viscosity surpasses that value, the resin is considered unusable. 

Figure 1 shows that both the reference resin and resin AC had poor stability, surpassing the viscosity limit after only eight days. Therefore, it can be inferred that the presence of DMeU in the aqueous medium does not contribute to stability. On the other hand, when the monomer is introduced in the condensation step, resin IC, viscosity remains stable below the 400 mPa∙s limit for more than two months, even at 40 °C. This suggests that DMeU is being effectively incorporated in the polymer structure, thus contributing to higher stability during storage.

To further support that DMeU is reacting with the polymer in the condensation step, the two resins containing the DMeU were analyzed by ^13^C NMR. The results are shown in Table 1. The assignment of the chemical shifts were made based in articles of UF resins [18,19] and articles with ^13^C NMR analysis of DMeU with formaldehyde [8,20]. The peaks present a little shift to the right due to the high DMSO-*d*_6_ content [18]. 

**Figure 2:**
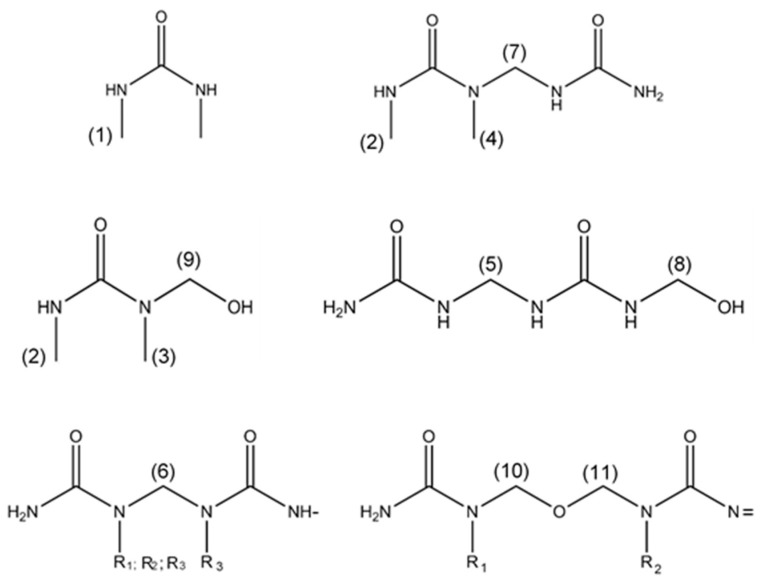
Chemical structures of molecules identified by ^13^C NMR in UF resins modified with DMeU. R_1_ = H, R_2_ = CH_2_OH; CH_2_-N=; CH_2_-O-CH_2_-N=; CH_3_, R_3_ = CH_2_OH; CH_2_-N=; CH_2_-O-CH_2_-N=.

Figure 2 illustrates the chemical structures observed in the ^13^C NMR spectra.

**Figure 3:**
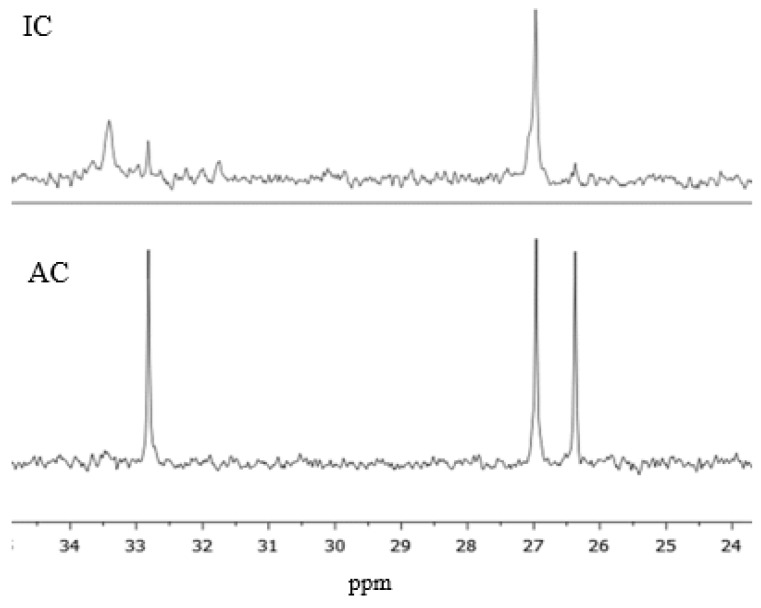
^13^C NMR spectrum of resins IC and AC.

Observing the chemical shifts presented in Table 1, it is possible to see a first group of peaks at lower chemical shift values, from 26 to 34 ppm, representing the dimethylurea derivatives. The peaks in this region can be seen in Figure 3, for both resins.

In Figure 3, the rightmost peak appears at 26.38 ppm, corresponding to the methyl carbon of a free DMeU (1). As expected, resin IC presents an almost imperceptible peak, at the level of the baseline noise, while resin AC shows a distinctive peak. This demonstrates the high degree of conversion of DMeU by reaction with formaldehyde and polymer when added during condensation. The next peak, at 26.97 ppm, corresponds to the methyl carbon on the opposite side of a hydroxymethyl group, or any type of linkage like methylene bridges, present in DMeU (2). The groups that contribute to this peak can result from methylolated DMeU or DMeU in an end group, which makes the analysis difficult. A peak at 32.82 ppm matches the methyl carbon of a methylolated DMeU linked to the same amino group as the hydroxymethyl group (3). There is a large difference between the two resins, with lower peak areas for resin IC. This indicates the existence of condensation reactions involving DMeU during the condensation step. Another peak appears at 33.41 ppm, which is related to the methyl carbon of a tertiary amine of DMeU linked to another monomer or polymer (4). This structure does not appear in resin AC, proving the absence of condensation of DMeU when added after the synthesis. As for resin IC, it presents a significant peak percentage for this type of linkage, representing DMeU groups in end groups. These two peaks at 32.83 ppm and 33.41 ppm allow to conclude that resin IC contains more DMeU linked to the polymer than in the methylolated form, and that resin AC only contains free DMeU and methylolated DMeU.

The next peaks of interest in the NMR analysis are related to linear and branched methylene bridges. Linear bridges are considered the ones that present only a linkage of an amino group of a urea with other monomers/polymers (secondary amino), while branched bridges are the ones that in addition to presenting that linkage, also contain another linkage in the amino group (tertiary amino), which could be a monomer/polymer or a hydroxymethyl group. 

The chemical shift at 45–46 ppm corresponds to linear methylene bridges (5). Resin IC shows slightly lower fraction of this structure. The peaks at 52–53 ppm are related to branched methylene bridges (6), where resin IC presents higher area percentages than resin AC. The presence of DMeU at the end groups limits the linear condensation of the polymer, since it is less reactive. That way it is forcing the reaction in other sites in the polymer structure, forming therefore more side groups like hydroxymethyl groups or polymer ramifications. Peaks at 53–54 ppm may be associated with a methylene bridge between one DMeU and one urea (7) [8]. Since this involves condensation reactions, the peak does not appear for resin AC, only for IC. When compared to the peak at 33.41 ppm it is possible to conclude that a significant amount, about 75%, should be DMeU linked to polymer end groups.

The following group of this analysis are the hydroxymethyl groups, more specifically, the carbon of the hydroxymethyl group, that could be linked to secondary amines (8) (63–64 ppm) or linked to tertiary amines (9) (68–70 ppm). In this case, resin IC presents a much lower gap between hydroxymethyl groups linked to secondary amines and linked to tertiary amines. This is a result of the presence of DMeU in the polymer. Since DMeU only has secondary amines, all hydroxymethyl groups linked to a DMeU will lead to tertiary amines (68–70 ppm). This reaction associated with the presence of the DMeU at end groups for resin IC, leads to a decrease of the available locations for the hydroxymethyl groups linked to primary aminos (63–64 ppm). Besides that, some DMeU of resin AC did not react with hydroxymethyl groups, neither monomers/polymers.

The peaks, at 67–68 and 72 ppm, correspond to linear (10) and branched (11) methylene-ether bridges, respectively. When comparing the two resins, resin AC presents higher area percentages of linear bridges, and resin IC higher area percentages of branched bridges. A possible reason for the significant difference of the area of branched methylene-ether bridges could be the reaction of monomethylolated DMeU with mono- or di-methylolurea, leading to a branched bridge, since DMeU will always have present the methyl group as side group. However, as explained above, in branched methylene bridges another phenomenon can occur, increasing the number of side groups and therefore the relative area of peaks pertaining to branched methylene ether bridges. 

As shown before, resin AC does not show evidence of condensation reactions, and therefore the methylene branched bridges are a consequence of methylolated urea with secondary amino groups.

The peak at 82 ppm matches the formaldehyde molecule, having a higher relative area in resin IC. This could be related to the contribution of DMeU in the condensation step, forming less hydroxymethyl groups, giving origin to lower consumption of formaldehyde. In Table 1 it is possible to observe the lower total content of hydroxymethyl groups in resin IC.

The last peaks correspond to carbonyl groups in urea and DMeU. Di-substituted urea appears at 157–158 ppm and monosubstituted urea at 158–159 ppm. The percentage areas are similar for both resins. It is not possible to take any conclusions from these values, because they are a mix of different carbonyl structures. The peak of free DMeU, at 159.5 ppm, is only present in resin AC, as noted before at 26.38 ppm, indicating full consumption of the monomer in resin IC. The absence of free urea is expected for both resin since the last urea is just added before particleboard production. 

### 3.2. Effect of DMeU Concentration 

The amount of DMeU added in the condensation step was decreased to 2.5% and 1.25% in order to evaluate its impact on storage stability. The addition of the DMeU was done at the beginning of the condensation step.

**Figure 4:**
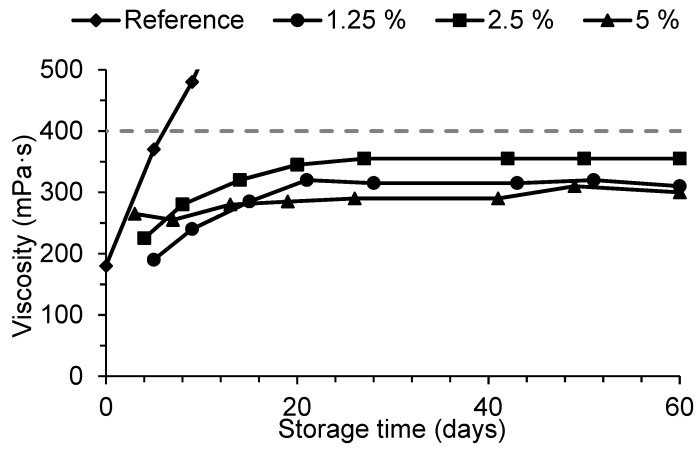
Viscosity as a function of storage time at 40 °C for reference (REF) resin and resins with addition of different amounts of dimethylurea at condensation step.

Figure 4 shows that storage stability was very similar for the three different amounts, with viscosity increasing initially and then stabilizing after 20–30 days. All resins were still stable after 60 days. The higher final viscosity of resin with 2.5% DMeU was related to the fact that viscosity at the beginning of the storage period was also slightly higher. Dimethylurea contents above 1.25% did not therefore seem to bring any gain, representing an unnecessary cost. These resins have also shown good stability when stored at 5 °C and 25 °C for two months. 

### 3.3. Physico-Mechanical Tests

After addition of the third urea, the resins’ adhesive performance was tested by ABES, which allows identifying the appropriate pressing time for wood particleboard production. Gel time measurements were also performed and are presented in Table 2.

**Figure 5:**
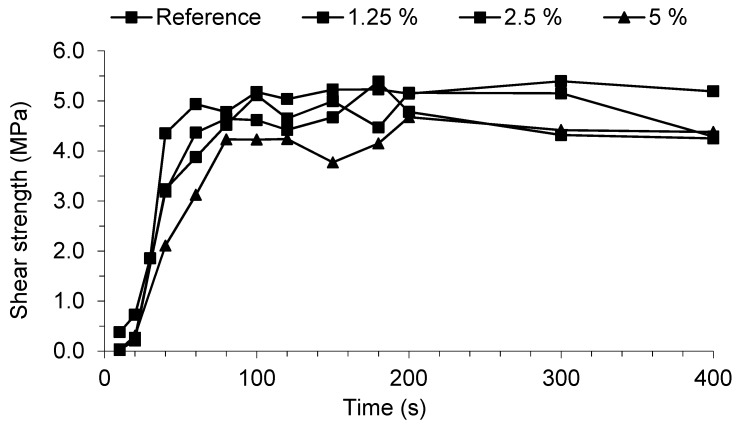
Analysis with an automated bonding evaluation system (ABES) technique at 105 °C of the REF resin and resins with dimethylurea before storage.

Figure 5 shows that the resins presented good shear strength values and good reactivity, achieving maximum shear strength between 80 and 100 s, and being close to the reference resin performance. However, between 100 and 120 s it is possible to see an increase in shear strength with decreasing DMeU content. 

Only at 5% DMeU content was the gel time significantly higher than for the reference resin (Table 2). This is in accordance with the lower initial slope observed in ABES results for this DMeU content.

The pressing time chosen for particleboard production was 120 s, since all resins reach maximum strength before that time, and it is the usual pressing time for commercial resin.

The particleboard panels were produced with these resins in different conditions: fresh, and after 1 and 2 months storage times.

The physico-mechanical properties of wood particleboards made with resins containing DMeU, shown in Table 3, are similar to those of the reference resin. Most importantly, performance did not tend to decrease along storage time, except for the resin with 5% DMeU. It seems that with the presence of higher amounts of DMeU, its lower reactivity becomes critical to the curing step.

In the fresh resins, the increase in DMeU percentage is associated with a decrease in formaldehyde content, since DMeU can react with free formaldehyde in the aqueous solution. This reduces formaldehyde content in the final panel, but may also contribute to less effective resin crosslinking. When comparing formaldehyde contents along storage time, a significant decrease is observed after two months. This is related to methylolation reactions being promoted by the basic pH and high temperature conditions during storage. It must be noted that all values of formaldehyde content were below the limit defined for E1 class particleboards, ≤8 mg/100 g oven dry board [21].

All panels also showed normal values for thickness swelling and moisture content, even for the resins applied after two months storage time.

## 4. Discussion and Conclusions

Adding DMeU to UF resin synthesis during the condensation step proved to be a successful strategy to attain two months of storage stability at 40 °C.

The ^13^C NMR analysis allowed some main conclusions:

- DMeU did not react with the polymer when it was added only at the end of the condensation step.
- About 75% of DMeU added to the resin during condensation reacted with the polymer.
- Incorporation of DMeU in the polymer resulted in a higher percentage of methylene and methylene-ether branched bridges.
- Virtually all DMeU reacted when it was added during condensation.

^13^C NMR analysis supported the hypothesis that incorporation of DMeU originates end groups with low reactivity during storage at basic pH, reducing the progress of condensation reactions, therefore improving dramatically the resin storage stability. An amount of 1.25% of DMeU was shown to be sufficient for this effect.

The mechanical properties of wood particleboards manufactured with fresh modified resins showed results equivalent to the reference resin. After storage at 40 °C for 1 or 2 months, the resins modified with 1.25% and 2.50% DMeU yielded internal bond strength values similar to the ones obtained with the fresh resins. Concerning formaldehyde content, the resins presented lower values than the reference resin, confirming the reaction of DMeU with formaldehyde. This value decreased further along storage time. 

The proposed strategy allowed the achievement of the goal of obtaining a very stable resin formulation under rather adverse temperature conditions, without impairing its physico-mechanical performance.

## Figures and Tables

**Table 1:** Chemical shifts and relative peak areas of methylene and carbonyl carbons in ^13^C NMR spectra of resins IC and AC.

Structure	Chemical Shift (ppm)	Relative Peak Area for Resin IC (%)	Relative Peak Area for Resin AC (%)
Methyl groups			
NH(CH_3_)–CO–NH(CH_3_) (1)	26.38	-	2.5
NH(CH_3_)–CO–N(CH_3_)– (2)	26.98	3.9	3.1
HO–N(CH_3_)–CO–NH(CH_3_) (3)	32.82	1.1	2.7
–N(CH_3_)–CO–NH(CH_3_) (4)	33.41	3.0	-
Methylene groups			
–NH–CH_2_–NH– (5)	45–46	6.4	7.0
–NH–CH_2_–N= (6)	51–53	10.5	9.0
–NH–CH_2_–N(CH_3_)– (7)	53–54	4.1	-
Hydroxymethyl groups			
–NH–CH_2_–OH (8)	63–64	11.9	14.3
=N–CH_2_–OH (9)	68–70	8.4	7.4
Methylene-ether groups			
–NH–CH_2_–O–CH_2_–NH– (10)	67–68	7.7	9.7
=N–CH_2_–O–CH_2_–NH– (11)	72	3.4	0.8
Formaldehyde			
HO–CH_2_–OH	82	1.6	1.1
Carbonyl groups			
H_2_N–CO–NH_2_	-	-	-
H_2_N–CO–NH–	158–159	21.7	21.3
=N–CO–NH–; –HN–CO–NH–	157–158	19.2	17.3
NH(CH_3_)–CO–NH(CH_3_)	159.5	-	1.4

**Table 2:** Gel time measurements for the reference resin and resins with different percentages of DMeU.

Resins	Reference	1.25%	2.5%	5%
Gel time (s)	54	59	59	67

**Table 3:** Physico-mechanical analysis of the particleboard produced with reference resin and resins modified with different percentages of dimethylurea, after different resin storage times.

Storage Time/Properties	Fresh				1 Month			2 Months		
DMeU %	REF	1.25%	2.5%	5%	1.25%	2.5%	5%	1.25%	2.5%	5%
Density (kg/m^3^)	667 ± 6	661 ± 8	660 ± 9	679 ± 7	656 ± 7	650 ± 8	645 ± 6	650 ± 7	670 ± 4	651 ± 8
Internal bond strength (N∙mm^−2^)	0.62 ± 0.01	0.54 ± 0.02	0.50 ± 0.02	0.51 ± 0.06	0.53 ± 0.05	0.52 ± 0.02	0.47 ± 0.04	0.52 ± 0.04	0.52 ± 0.06	0.43 ± 0.05
Thickness swelling (%)	43.7 ± 0.9	37.1 ± 1.1	37.1 ± 1.1	42.7 ± 3.3	35.4 ± 2.1	32.8 ± 0.8	39.8 ± 1.1	36.7 ± 1.3	36.7 ± 1.8	37.6 ± 3.1
Moisture content (%)	6.5 ± 0.5	7.1 ±.5	6.8 ± 0.2	6.5 ± 0.5	5.5 ± 0.1	5.4 ± 0.1	5.6 ± 0.1	6.4 ± 0.1	7.5 ± 0.2	6.4 ± 0.4
Formaldehyde content (mg/100 g oven dry board)	7.9	6.9	6.5	5.9	-	-	-	5.6	5.5	5.4
